# Review of Temperature Management in Traumatic Brain Injuries

**DOI:** 10.3390/jcm13072144

**Published:** 2024-04-08

**Authors:** Kenya Kawakita, Hajime Shishido, Yasuhiro Kuroda

**Affiliations:** 1 Emergency Medical Center, Kagawa University Hospital, Miki 761-0793, Japan; ranran.med.kagawa@gmail.com; 2 Department of Emergency, Disaster, and Critical Care Medicine, Faculty of Medicine, Kagawa University, Miki 760-0793, Japan; kuroda.yasuhiro@kagawa-u.ac.jp

**Keywords:** traumatic brain injury, targeted temperature management, neurointensive care

## Abstract

Therapeutic hypothermia (TH) for severe traumatic brain injury has seen restricted application due to the outcomes of randomized controlled trials (RCTs) conducted since 2000. In contrast with earlier RCTs, recent trials have implemented active normothermia management in control groups, ensuring comparable intensities of non-temperature-related therapeutic interventions, such as neurointensive care. This change in approach may be a contributing factor to the inability to establish the efficacy of TH. Currently, an active temperature management method using temperature control devices is termed “targeted temperature management (TTM)”. One of the goals of TTM for severe traumatic brain injury is the regulation of increased intracranial pressure, employing TTM as a methodology for intracranial pressure management. Additionally, fever in traumatic brain injury has been acknowledged as contributing to poor prognosis, underscoring the importance of proactively preventing fever. TTM is also employed for the preemptive prevention of fever in severe traumatic brain injury. As an integral component of current neurointensive care, it is crucial to precisely delineate the targets of TTM and to potentially apply them in the treatment of severe traumatic brain injury.

## 1. Introduction

The concept of targeted temperature management (TTM) has undergone significant development since the 1990s. Initially, TTM primarily referred to therapeutic hypothermia (TH), with a focus on proactively applying TH, in the belief that it improves neurological outcomes in cases of severe traumatic brain injury. In fact, individuals with severe traumatic brain injury who underwent TH regained consciousness and eventually resumed their daily lives following intensive care. Multiple clinical studies [1,2,3,4] have provided evidence of the efficacy of TH, prompting its adoption at our institution for cases of severe traumatic brain injury. However, since 2000, randomized controlled trials (RCTs) such as NABIS:H [5] have emerged, prompting the reevaluation of the effectiveness of TH [6,7]. The aim of this review is to evaluate the efficacy of TH for traumatic brain injury and to propose strategies for the utilization of TTM. In keeping with the Dasic matrix [8] for the unmet needs of neurotrauma services in major trauma centers, this article intends to appraise the evidence on the use of TTM in severe traumatic brain injury.

## 2. Materials and Methods

A narrative review of the efficacy of TH and strategies for the utilization of TTM in patients with traumatic brain injury was conducted. PubMed and ClinicalTrials.gov databases were searched using the following search (MeSH) terms: “head trauma”, “head injury”, “traumatic brain injury”, “therapeutic hypothermia”, and “hypothermia” with the Boolean operators “AND” and “OR”. Considering recent advances in intensive care, we focused only on RCTs published 1 January 2000, and 31 December 2022. Furthermore, given the nature of this review, only relatively large RCTs with 80 or more participants were considered. However, the most recent RCT in which we participated, HOPSE trial [9], was included in this review. All identified studies were summarized and the target temperature, actual control temperature and methods of temperature management in the control group were reviewed.

## 3. Historical Background

Clifton et al. conducted research that compared the neurological outcomes and mortality rates after 6 months in two groups of patients with severe traumatic brain injuries. One group consisted of 190 patients who received TH, aiming to lower their core temperature to 33 °C within 8 h of injury and maintain it 48 h. The other group, serving as the control, comprised 178 patients who did not undergo active temperature management. While no significant differences were observed overall, a subgroup analysis showed favorable outcomes at 6 months for the hypothermia group among patients with an initial temperature of 35 °C and who were aged 45 years [5]. Therefore, the NABIS:H2 study narrowed the comparison to patients aged 45 years and younger who started treatment within 2.5 h of injury. However, when subgroup analysis was conducted among patients undergoing hematoma evacuation, it was found that the hypothermia group exhibited favorable neurological outcomes at the 6-month mark [7]. Moreover, Clifton et al. conducted a post hoc analysis involving 489 patients by combining data from both the NABIS:H and NABIS:H2 studies. This analysis demonstrated the potential for enhancing neurological outcomes by implementing hypothermia at 35 °C both before and after hematoma removal, followed by maintaining a temperature of 33 °C for 48 h [10]. Building upon these findings, Hergenroeder et al. compared the 6-month neurological outcomes between hypothermia and control groups in patients with acute subdural hematoma possessing a Glasgow coma scale (GCS) motor score of 5 and qualifying for surgical intervention. In the hypothermia group, the core body temperature was reduced to 35 °C before dura incision and then maintained at 33 °C for 48 h. A distinctive feature of this study was the strict temperature control achieved in both groups through intravascular cooling catheters. However, the study was discontinued after an interim analysis of 32 patients indicated that achieving the study objectives was improbable [9]. The results underscore the challenges associated with the conducting of an RCT on hypothermia therapy for severe traumatic brain injury. Moreover, Maekawa et al. compared 140 patients with severe traumatic brain injury and a GCS score of 4–8 who underwent hypothermia therapy at 32–34 °C for 72 h with a control group whose temperatures were maintained at 35.5–37 °C. They found no significant differences in neurological outcomes or mortality rates at 6 months between the two groups [6].

Subsequent meta-analyses and studies have intermittently highlighted the effectiveness of therapeutic hypothermia [11,12,13]. However, the data on the overall efficacy of TH for severe traumatic brain injury is still limited. Although present, clinical studies assessing the efficacy of TH for traumatic brain injury are of low quality, potentially contributing to the absence of consensus in meta-analyses. The guidelines in the United States [14] do not recommend TH for diffuse brain injury, although there is a suggestion that it may be effective when hematoma evacuation is performed [15].

Similarly, for post-cardiac arrest syndrome (PCAS), a series of RCTs demonstrating the efficacy of TH on neurological outcomes were reported during the 2000s [16,17]. In response to these findings, the 2010 American Heart Association (AHA) guidelines [18] strongly recommended TH in the range of 32–34 °C for patients with PCAS after out-of-hospital cardiac arrest due to ventricular fibrillation who had achieved the return of spontaneous circulation. However, in 2013, an RCT comparing groups managed at 36 °C and 33 °C found no significant difference in neurological outcomes [19]. Consequently, AHA guidelines [20] since 2015 have not specified a target temperature for managing patients with PCAS, instead recommending TTM within the range of 32–36 °C. The findings from the TTM2 trial [21], published in 2021, indicate no significant difference in the 6-month mortality rate and neurological outcomes between patients undergoing TH at 33 °C and those exposed to aggressive normothermia. In the 2022 International Consensus Conference on Cardiopulmonary Resuscitation and Emergency Cardiovascular Care Science with Treatment Recommendations (CoSTR) [22], hosted by the International Liaison Committee On Resuscitation (ILCOR), a suggestion was put forth advocating for actively preventing fever (APF) with a target temperature not exceeding 37.5 °C in patients enduring persistent coma following the return of spontaneous circulation in adult cardiac arrest. Furthermore, for patients with persistent coma, it is recommended to maintain APF for a minimum duration of 72 h. In the context of PCAS, there has been a trend toward adopting higher target temperatures for TTM. As TH, which has shown effectiveness in animal models, is applied to human patients, proving its efficacy has become increasingly difficult due to various biases and patient-related factors. Even within the relatively homogenized population of PCAS, recent RCTs have failed to establish the effectiveness of TH. Consequently, if TH cannot be demonstrated as being effective in PCAS, a condition with relatively consistent characteristics, proving its efficacy in more complex pathophysiological conditions like severe traumatic brain injury becomes even more challenging.

This has contributed to a shifting perspective that considers that the aggressive lowering and controlling of body temperature in patients with severe brain damage may not necessarily result in improved neurological outcomes. Nevertheless, the response to the question of whether temperature management is dispensable in neurointensive care is “no.” TTM within neurointensive care serves as a critical element in strategies designed to mitigate secondary brain injury in patients with acute brain disorders. TTM remains a crucial component in neurocritical care.

In animal experiments, mild hypothermia has been demonstrated to inhibit apoptosis of newly generated cells in the hippocampus and endoplasmic reticulum stress-induced apoptosis, ultimately suppressing TBI-induced cognitive impairments [23,24]. Moreover, mild hypothermia has been shown to suppress the exacerbated blood–brain barrier permeability and potentially reduce brain edema [25].

**Figure 1:**
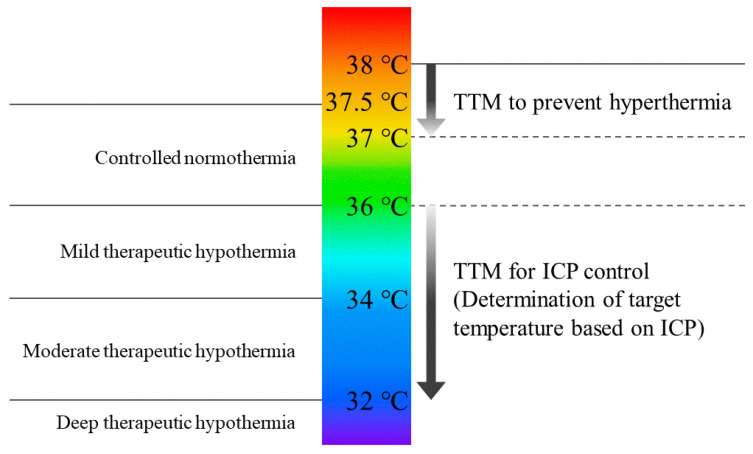
Changes in temperature management methods for severe traumatic brain injury. On the left side, conventional therapeutic hypothermia is depicted, while on the right side, the concept of TTM for severe TBI is presented. TBI, traumatic brain injury; TTM, targeted temperature management.

Within our facility, TTM serves two primary purposes: (1) TTM for the regulation of intracranial pressure (ICP) and (2) TTM for the prevention of hyperthermia (Figure 1). Furthermore, we have integrated TTM into our protocols for PCAS and heat-related illnesses (Table 1). The focus of ICP control primarily centers on severe traumatic brain injury and stroke cases, with the aim of maintaining a core temperature within the range of 33–36 °C, adjusted according to ICP levels. Utilized temperature management devices encompass Thermogard System endovascular cooling using catheters (Zoll, Sunnyvale, CA, USA) or Arctic Sun gel-coated adhesive pads (Bard Medical, St. Louis, CO, USA). Adequate sedation and analgesia are administered, with TTM typically spanning approximately 1 week, although in severe traumatic brain injury cases, it may extend up to 2 weeks. Conditions targeted for fever prevention include severe traumatic brain injury and stroke, predominantly subarachnoid hemorrhage cases. The target temperature range is set at 37–38 °C, commonly employing intravascular cooling catheters. This method offers the advantage of applicability even during consciousness; however, measures to mitigate shivering are imperative. TTM is also instituted for PCAS patients, with the target temperature range spanning 32–36 °C, allowing for flexibility based on the patient’s hemodynamic condition. In instances of heatstroke accompanied by loss of consciousness, rapid reduction of body temperature is crucial, employing intravascular cooling devices. Once the target temperature is achieved, early discontinuation of TTM is feasible.

## 4. Terminology of Body Temperature Management

The terminology related to TH lacked consistency among researchers until Polderman et al. organized and proposed a classification in 2009 [26]. Maintaining core body temperature within the range of 34.0–35.9 °C is categorized as mild TH, while temperatures between 32.0 and 33.9 °C are classified as moderate TH, and temperatures < 31.9 °C are considered deep TH. Additionally, the method of controlling and maintaining temperatures between 36.0 and 37.5 °C, along with addressing shivering, is termed “controlled normothermia”. In recent years, the term “TTM” has become more prevalent over TH. TTM involves utilizing a device to regulate body temperature and encompasses shivering management. As shivering can occur even at 37 °C [27], it is appropriate to designate active control of core body temperature < 37.5 °C as TTM. In our institution, the term “anti-hyperthermia” is used for the application of antipyretic agents and simple surface cooling measures for fever without employing temperature management devices. The ILCOR recommends avoiding the term “TTM” in order to prevent confusion with the TTM trial. Instead, terms, such as “temperature control with hypothermia”, “temperature control with normothermia”, and “fever prevention”, are recommended [28]. However, for convenience, the term “TTM” is utilized in this text. In the future, there is a hope to develop terminology tailored to the purpose and therapeutic intensity of TTM.

## 5. TTM for ICP Control

Numerous studies have shown that lowering body temperature results in a reduction in ICP [1,5,29,30,31,32], and this correlation is well-documented empirically. Furthermore, for every 1 °C decrease in body temperature, there is typically a corresponding 7–8% decline in cerebral blood flow and cerebral oxygen consumption [33]. This highlights the potential of hypothermia, such as TTM, to regulate the balance between cerebral oxygen metabolism supply and demand. When hypothermia is pursued with the goal of improving patient neurological outcomes, its application may be limited to specific cases. However, when the primary treatment objective shifts to ICP control, the range of cases with elevated ICP that can benefit from hypothermia widens. TTM, rather than being employed solely for prevention, can also serve a therapeutic role [11]. Successful ICP management through TTM not only facilitates life-saving interventions but also sets the stage for subsequent rehabilitation efforts.

The Seattle Consensus [34] recommends a stepwise escalation of intensive care strategies for managing severe traumatic brain injury. This approach includes the most intensive treatments, such as TH, barbiturate therapy, and decompressive craniectomy. The fundamental approach outlined in the Seattle Consensus emphasizes measures likes elevating the head, providing appropriate sedation and analgesia, and mechanical ventilation, irrespective of intracranial pressure levels. Additional measures include administering anticonvulsants within the first week, maintaining hemoglobin levels > 7 g/dL, ensuring SpO_2_ remains > 94%, and preventing hyponatremia. Initial interventions for elevated ICP involve intensified sedation, analgesia, the use of osmotic diuretics, external ventricular drainage, and mild hyperventilation therapy. As a secondary measure, moderate hyperventilation therapy and the use of muscle relaxants are recommended. At this stage, performing a mean arterial pressure (MAP) challenge is advised in order to assess whether traumatic brain injury has compromised cerebral autoregulation (CA). This involves administering a vasopressor to increase MAP by 10 mmHg for a brief period and observing the resulting change in ICP. If ICP increases with increasing MAP, CA may be impaired. Conversely, if ICP decreases with increasing MAP, CA is considered intact. If CA is preserved, ICP may be managed through fluid loading or the use of vasopressors. The third step involves considerations regarding TH, barbiturate therapy, and decompressive craniectomy. Although there is an ongoing debate about the initial choice of method during this phase, there is a consensus around the need to prioritize craniectomy without hesitation in cases of an expanding lesion with a gradual increase in ICP over time. It is important to recognize that, while TH and barbiturate therapy are medical interventions, they inherently have limitations in controlling ICP. In an RCT conducted by Hui et al. [35] involving 302 cases of severe traumatic brain injury, subgroup analysis revealed that, in patients who underwent decompressive craniectomy, there was no significant difference in ICP values during the treatment period between the two groups. However, the study demonstrated the potential utility of TH in patients not undergoing decompressive craniectomy for managing ICP. Essentially, TTM might be effective when ICP control through medical intervention is anticipated. Furthermore, in cases of elevated ICP following decompressive craniectomy, considering TTM as one of the treatment options would be deemed acceptable. Barbiturate therapy is known to induce circulatory depression and immunosuppression, and its use requires expertise. Leger et al. allocated 383 patients with head trauma showing intracranial pressure elevation into two groups based on the use or absence of barbiturate therapy and reported the treatment outcomes. This study found an association between the early introduction of barbiturate therapy and increased ICU mortality. However, differences between the two groups in the incidence of ventilator-associated pneumonia or neurological outcomes at 3 months were undetected [36]. It is important to note that barbiturates can also be used as a final step in managing patients with elevated ICP, despite their potential side effects. Kajiwara et al., conversely, demonstrated the efficacy of step-down infusion barbiturate therapy, gradually reducing the dose of thiopental from 3.0 mg/kg/h to 1.0 mg/kg/h, combined with normothermia therapy in terms of mortality and ICP control. They have also reported that complications specific to barbiturate therapy did not impact outcomes and were manageable [37]. This study indicates new possibilities for combination therapy with TH and barbiturate therapy.

When employing TTM for ICP control in our institution, we utilize deep sedation and muscle relaxants, along with either vascular cooling catheters (Thermogard System^®^, Zoll, Sunnyvale, CA, USA) or water pads (Arctic Sun^®^, Bard Medical, St. Louis, CO, USA). This combination is employed to achieve controlled deep body temperatures within the range of 33–36 °C. Our target patient population encompasses various cases, including severe traumatic brain injury, severe subarachnoid hemorrhage, cerebral hemorrhage, and extensive cerebral infarction. TTM is employed to circumvent the need for craniotomy and to address elevated ICP post-craniotomy. The initial setting for the target temperature aims for a value close to 36 °C, taking into account previous ICP measurements and CT findings. If the ICP at the time that the core body temperature reaches the target temperature is deemed insufficient, a further reduction of the target temperature is pursued. While some approaches advocate setting the initial target temperature lower, such as 33 °C, we opt for a step-down approach based on ICP. This allows for effective ICP control at near-normal core body temperatures without excessively lowering the body temperature. This approach’s advantage lies in the potential for effective ICP control at 36 °C, with a small temperature difference from normothermia, thereby reducing the risk of complications, such as pneumonia, coagulation disorder and electrolyte imbalance, associated with hypothermia and facilitating relatively straightforward rewarming. However, a drawback is that setting the target temperature often relies on the subjective judgment of the attending physician, presenting challenges in standardizing temperature-setting protocols.

## 6. TTM to Prevent Hyperthermia

In the context of infectious diseases, fever serves as a systemic response aimed at eliminating bacteria and viruses from the body. Therefore, there is ongoing debate regarding the necessity of employing antipyretic measures. When fever is the sole symptom without accompanying manifestations, it is common practice to monitor the course without resorting to antipyretic treatment. However, the situation is different for patients with severe traumatic brain injury. Fever is a frequently observed systemic response in patients with severe brain disorders, and its association with neurological outcomes has been noted in various conditions, including head trauma, stroke, and PCAS [38,39,40,41]. Fever is recognized as a factor that can worsen neurological outcomes. In cases of head trauma, it has been noted that inflammatory cytokines in damaged tissues and the elevation of excitatory amino acids are mitigated by lowering body temperature [42,43]. Small elevation of brain temperature in ischemic and injured brain is thought to exacerbate pathological changes and promote early disruption of the blood–brain barrier [44]. Furthermore, it has been found that moderate hypothermia reduces the vascular permeability of the blood–brain barrier in injured brain [45]. Therefore, therapeutic intervention may be necessary for patients with severe brain injury, depending on the presence of fever. Critical questions arise regarding the determination of the temperature threshold at which intervention should begin and the identification of the methods to be employed for fever reduction. Currently, there are no definitive answers to these questions. Additionally, the duration of fever management is also an important consideration. IL-1-mediated inflammatory responses have been reported to persist for up to 3 weeks after traumatic brain injury [46], suggesting that, contrary to previous belief, longer-term temperature management may be necessary.

In cases of severe traumatic brain injury, basic care in neurointensive care follows the guidelines outlined in the Seattle Consensus [34], with the aim of maintaining core body temperature < 38 °C. Picetti et al. conducted a survey among intensive care physicians in Europe, reporting that fever was defined as a body temperature > 38 °C, and that treatment thresholds are therefore set by physicians at these temperatures [47]. This value is considered appropriate. Initially, attempts have been made to reduce fever through three-point cooling (neck, axilla, and groin) and via the administration of antipyretic medications. However, if antipyretic medications prove ineffective or lead to excessive hypotension, TTM is initiated using intravascular cooling catheters or water pads while the patient is under sedation and analgesia.

Here, our focus lies on the control group of RCTs investigating the efficacy of TH for severe traumatic brain injury. It is assumed that appropriate intensive care was provided in the TH intervention group during the RCT. However, our attention is directed toward understanding how intensive care was administered in the control group. Andrews et al. conducted the Eurotherm3235 trial [48], where TH was applied at 32–35 °C for more than 48 h in 387 patients with traumatic head injuries experiencing ICP exceeding 20 mmHg within 10 days post-injury. This study compared neurological outcomes and mortality at 6 months between a group receiving standard care only (control group) and an intervention group. The results indicate superior outcomes in both aspects for the control group. A notable aspect of this clinical study was the core body temperature observed in the control group, which, in many cases, was maintained at approximately 37 °C. In 2018, Cooper et al. presented the results of the POLAR study [49]. This clinical research aimed to initiate TH early in 495 patients with a GCS score of ≤ 8. Patient enrollment began before arrival at the hospital, with the aim of administering hypothermia as soon as possible. This clinical study compared neurological outcomes and mortality rates at 6 months between the hypothermia group, which underwent TH at 33–35 °C for at least 72 h and was then gradually re-warmed at a maximum rate of 0.25 °C/h, and the normothermia group, which actively managed to maintain a temperature of 37.0 °C ± 0.5 °C. No significant differences were observed in the outcomes between the two groups. Table 2 presents RCTs that did not establish the effectiveness of TH. In numerous studies, it is evident that strict temperature control was implemented using temperature control devices in the control group. Furthermore, a significant portion of these control groups maintained a target temperature of 37 °C, indicating management strategies aimed at preventing fever. Conversely, Table 3 presents RCTs that demonstrated the effectiveness of TH. The approach to temperature management in the control groups of these RCTs was either not actively implemented or remains unclear. Watson et al. also addressed temperature management in the control group, noting that studies where temperature control was either not implemented or where temperatures were maintained at >38 °C, even if managed, and where the methods were unclear, exhibited higher mortality rates. Conversely, studies where temperature management was implemented in the control group at <38 °C showed comparable mortality rates to TH [12]. From these observations, it can be inferred that, in addition to standard care, actively suppressing fever, rather than lowering body temperature, may contribute to improved neurological outcomes in patients with traumatic head injuries. In neurointensive care, it is crucial to consider not only the patient’s temperature but also various other aspects of the disease’s pathology, including management of respiratory and circulatory functions, and monitoring and treatment of ICP, CPP, blood glucose, electrolytes, nutrition, and other factors. Furthermore, prevention, assessment, and treatment of complications, such as acute symptomatic seizures, systemic infections, and venous thromboembolism, are essential components of neurointensive care and must be carried out meticulously.

## 7. Prevention of Shivering during TTM

During TTM, careful attention is required to address shivering, which is a physiological homeostatic response that usually initiates at approximately 35.5 °C. Shivering not only disrupts the desired temperature control but also increases cerebral metabolism and may contribute to elevated ICP. Preventing secondary brain injury caused by shivering is crucial during TTM. Shivering assessment typically begins using the bedside shivering assessment scale (BSAS) [51]. In some cases, electromyographic interference on electrocardiography is recognized as an indication to initiate shivering interventions. Shivering protocols often involve a stepwise approach [52,53]. In the first step, interventions include antipyretic analgesics, such as acetaminophen, magnesium preparations, and counter warming, of the upper limbs and neck. The second step involves the use of medications, such as meperidine, fentanyl, and dexmedetomidine. The third step includes bolus administration and titration of medications, such as propofol and midazolam, and as a last resort, muscle relaxants may be used, provided adequate sedation is maintained (Table 4).

## 8. Limitations

We have emphasized the importance of defining the purpose of TTM and ensuring high-quality neurointensive care for effective implementation. The primary aim of TTM is ICP control. Although TTM may reduce ICP, its impact on the improvement of neurological outcomes in patients with traumatic brain injury remains uncertain. Lowering ICP could potentially prevent fatal secondary brain injuries, but the significance of TTM may diminish if patients experience neurological disabilities or prolonged consciousness disorders post-resuscitation. Nonetheless, frontline emergency physicians, neurosurgeons, and neurointensive care specialists prioritize saving lives as their primary treatment objective. Upon achieving survival, post-acute rehabilitation efforts may aid in recovering impaired neurological functions. However, it is crucial to investigate whether ICP control through TTM can enhance the long-term neurological outcomes of patients with traumatic brain injury.

The second purpose of TTM is fever prevention. Presently, the Seattle Consensus recommends maintaining a temperature of ≤ 38 °C after severe TBI; however, a definitive standard for the target temperature is currently unavailable, and the optimal method for reducing temperature remains unclear. Current options include regular administration of antipyretic analgesics and the use of temperature management devices. While regular administration of antipyretic analgesics is convenient, it has drawbacks, such as hypotension and challenges in maintaining strict temperature control. Conversely, using temperature management devices allows for more precise temperature control but may lead to secondary brain damage, such as shivering, necessitating new treatment approaches. Furthermore, the duration of fever prevention post-traumatic brain injury remains uncertain, warranting investigation into prevention methods and the appropriate duration of continuation.

Coagulopathy in traumatic brain injury has been associated with increased morbidity and mortality [54]. Additionally, there is concern that TH may exacerbate coagulopathy. Post hoc analyses of the BHYPO study [6] and the POLAR trial [49] were conducted. The safety of TH in traumatic brain injury patients with coagulopathy was evaluated using activated partial thromboplastin time (APTT) and fibrin/fibrinogen degradation product (FDP). Although a significant prolongation of APTT was observed in the TH group, there were no differences in patient outcomes [55]. Furthermore, the effect of therapeutic hypothermia on clot formation using thromboelastography was not in a range that would have clinical implications [56]. Both studies controlled TH group at a target temperature of 33 °C, suggesting that TTM up to 33 °C can be safety implemented in patients with coagulopathy following traumatic brain injury.

## 9. Conclusions

In recent years, there have been remarkable advancements in intensive care, surpassing TH. When employing TTM, it is crucial to establish explicit objectives of TTM. In addition to lowering body temperature, our focus is on delivering thorough and comprehensive care, which includes meticulous attention to respiratory management, circulatory support, shivering control, electrolyte balance, nutrition, blood glucose control, prevention and treatment of acute symptomatic seizures and systemic complications, and the addressing of other relevant aspects of patient care. A TTM approach of high quality, grounded in the understanding of minimizing secondary brain injury, is thought to play a role in fostering favorable patient outcomes beyond the mere reduction of body temperature. Neurointensive care requires collaboration among multiple disciplines as it cannot be performed by emergency physician or intensive care physician alone.

## 10. Future Directions

As neurointensive care for severe traumatic brain injury progresses, various neuro-monitoring techniques beyond temperature management are being employed. Robba et al. have demonstrated that the use of ICP monitoring allows for increased treatment intensity at the appropriate time, leading to improved long-term outcomes for traumatic brain injury patients [57]. In contemporary neurointensive care, multimodality monitoring has become essential. Contrast-enhanced near-infrared spectroscopy (NIRS) with indocyanine green (ICG) is considered a suitable neuromonitoring tool as it has the potential to assess cerebral blood flow in a minimally invasive manner [58]. CA has garnered attention, particularly concerning ICP and CPP. Through the continuous monitoring of variables, such as ICP and MAP, it becomes possible to assess CA impairment directly at the bedside. The degree of impairment in CA is thought to be associated with neurological outcomes after traumatic brain injury. Exploring the correlation between temperature and CA is an intriguing area of research, as is examining whether temperature fluctuations influence CA and whether temperature management can impact CA. Moreover, there is anticipation for studies investigating whether maintaining optimal body temperature, blood pressure, and CPP can positively influence outcomes for patients with severe traumatic brain injury.

## Figures and Tables

**Table 1:** Example of TTM used at our institution.

Purpose	ICP Control	Prevention of Fever	Prevention of Secondary Brain Damage in PCAS	Reducing Fever in Heat Patients with Stroke
Subject	Severe TBI and stroke	Severe TBI and stroke	PCAS	Severe heat stroke
Target temperature	33–36 °C	37–38 °C	32–36 °C	38 °C
Device	Intravascular catheters Surface cooling	Intravascular catheters *	Intravascular catheters Surface cooling	Intravascular catheters
Medication	Analgosedative drugs, muscle relaxants, and antipyretic drugs	Antipyretic drugs and analgosedative drugs	Analgosedative drugs, muscle relaxants, and antipyretic drugs	Analgosedative drugs and muscle relaxants
Respirator	with	with or without	with	with
Duration	1–2 weeks	1–2 weeks	~1 week	1–2 days

TTM, targeted temperature management; ICP, intracranial pressure; PCAS, post-cardiac arrest syndrome; TBI, traumatic brain injury. * When temperature control is not possible with antipyretic drugs.

**Table 2:** Characteristics of the major randomized controlled trials that failed to demonstrate the effectiveness of therapeutic hypothermia in severe traumatic brain injury.

Study	Participants (Hypothermia/Control)	Target Temperature (°C) (Hypothermia/Control)	Mean Temperature (°C) of the Control Group	Temperature Management Device (Hypothermia/Control)	Cooling Methods of the Hypothermia Group	Poor Outcome (Hypothermia/Control), *p*-Value
Shiozaki 2001 [1]	45/46	33.5–34.5/36.5–37.5	n.a.	Cooling blankets/surface cooling	Cooling blankets, gastric lavage with cold fluid	53%/41%, *p* = 0.251
Clifton 2001 NABIS: H [5]	190/178	32.5–34.0/37.0	37.2 ± 0.8	Temperature control pads/n.a.	Gastric lavage with cold fluid and room temperature ventilated air	57%/57%, *p* = 0.99
Clifton 2011 NABIS: H2 [7]	52/45	33.0/37.0	37.2 ± 0.4	Surface cooling (Arctic Sun^®^)/cooling blankets	Intravenous cold fluid, gastric lavage with cold fluid, surface cooling, room temperature of ventilated air	60%/56%, *p* = 0.67
Maekawa 2015 BHYPO [6]	95/45	32.0–34.0/35.5–37.0	35.7 ± 1.0 (Day 1)	Cooling blankets/cooling blankets	Surface cooling, gastric lavage with cold fluid and intravenous cold fluid	53%/48%, *p* = 0.597
Andrews 2015 Eurotherm3235 [48]	195/192	32.0–35.0/n. a.	37.0	Usual cooling technique/n.a.	Intravenous cold fluid	74.3%/63.5%, *p* = 0.03
Cooper 2018 POLAR [49]	256/239	32.5–33.5/36.5–37.5	37.0	Surface cooling/surface cooling	Intravenous cold fluid and surface cooling	51.2%/49.1%, *p* = 0.94
Hui 2021 LTH-1 [35]	156/146	34.0–35.0/37.0	37.08	Cooling blankets/surface cooling	Cooling blankets	41.3%/51.9%, *p* = 0.105
Hergenroeder 2022 HOPES [9]	16/16	33.0/37.0	36.0–37.0	Intravascular catheters (Thermogard System^®^)/intravascular catheters (Thermogard System^®^)	Intravascular cooling	62%/75%, *p* = 0.35

n.a.—not applicable.

**Table 3:** Characteristics of major randomized controlled trials that have established the effectiveness of therapeutic hypothermia in severe traumatic brain injury.

Study	Participants (Hypothermia/Control)	Target Temperature (°C) (Hypothermia/Control)	Mean Temperature (°C) of the Control Group	Temperature Management Device (Hypothermia/Control)	Cooling Methods of the Hypothermia Group	Poor Outcome (Hypothermia/Control), *p*-Value
Jiang 2000 [2]	43/44	33–35/37–38	n.a.	Cooling blankets/n.a.	Cooling blankets	53.5%/72.7%, *p* < 0.05
Qiu 2007 [31]	40/40	34.5–36.0/37.5–38.0	n.a.	Cooling blankets, cooling caps/none	Cooling blankets, cooling caps, surface cooling, intravenous cold fluid	30.0%/52.5%, *p* = 0.041
Zhi 2003 [32]	198/198	32.0–35.0/36.5–37.0	n.a.	Cooling blankets/n.a.	Cooling blankets	38.3%/62.1%, *p*: n.a.
Zhao 2011 [50]	40/41	32.5–33.0/37.0	n.a.	Cooling blankets/n.a.	Cooling blankets	25.0%/48.8%, *p* = 0.038

n.a.—not applicable.

**Table 4:** Example of shivering protocol for TTM at our institution.

STEP 1 (Go to next step if BSAS > 0)	Acetaminophen 650–1000 mg IV/PO q 4–6 h Skin counter-warming Magnesium sulfate 20 mEq/20 mg IV (if with hypomagnesemia)
STEP 2 (Go to next step if BSAS > 0)	Meperidine 35–50 mg IV q 3–4 h and/or Dexmedetomidine IV infusion 0.2 mcg/kg/h and/or Fentanyl IV infusion 25 mcg/h
STEP 3 (Go to next step if BSAS > 0)	Intubation if not already undertaken Propofol 20–40 mg IV bolus followed by 0.5–3 mg/kg/h or Midazolam 3–5 mg IV bolus followed by 0.06–0.18 mg/kg/h
STEP 4	Rocuronium 50 mg IV bolus followed by 10–20 mg/h

TTM, targeted temperature management; BSAS, bedside shivering assessment scale; IV, intravenous; PO, per oral.

## Data Availability

Not applicable.

## References

[1] Shiozaki T., Hayakata T., Taneda M., Nakajima Y. (2001). A Multicenter Prospective Randomized Controlled Trial of the Efficacy of Mild Hypothermia for Severely Head Injured Patients with Low Intracranial Pressure. J. Neurosurg..

[2] Jiang J., Yu M., Zhu C. (2000). Effect of Long-Term Mild Hypothermia Therapy in Patients with Severe Traumatic Brain Injury: 1-Year Follow-up Review of 87 Cases. J. Neurosurg..

[3] Marion D.W., Penrod L.E., Kelsey S.F., Obrist W.D. (1997). Treatment of Traumatic Brain Injury with Moderate Hypothermia. N. Engl. J. Med..

[4] Clifton G.L., Allen S., Barrodale P., Plenger P. (1993). A Phase II Study of Moderate Hypothermia in Severe Brain Injury. J. Neurotrauma.

[5] Clifton G.L., Miller E.R., Sung C.C., Levin H.S. (2001). Lack of Effect of Induction of Hypothermia after Acute Brain Injury. N. Engl. J. Med..

[6] Maekawa T., Yamashita S., Nagao S., Hayashi N., Ohashi Y., Aibiki M., Aruga T., Asai Y., Dohi K., Eguchi Y. (2015). Prolonged Mild Therapeutic Hypothermia versus Fever Control with Tight Hemodynamic Monitoring and Slow Rewarming in Patients with Severe Traumatic Brain Injury: A Randomized Controlled Trial. J. Neurotrauma.

[7] Clifton G.L., Valadka A., Zygun D., Coffey C.S. (2011). Very Early Hypothermia Induction in Patients with Severe Brain Injury (the National Acute Brain Injury Study: Hypothermia II): A Randomised Trial. Lancet Neurol..

[8] Dasic D., Morgan L., Panezai A., Syrmos N., Ligarotti G.K.I., Zaed I., Chibbaro S., Khan T., Prisco L., Ganau M. (2022). A Scoping Review on the Challenges, Improvement Programs, and Relevant Output Metrics for Neurotrauma Services in Major Trauma Centers. Surg. Neurol. Int..

[9] Hergenroeder G.W., Yokobori S., Choi H.A., Schmitt K., Detry M.A., Schmitt L.H., McGlothlin A., Puccio A.M., Jagid J., Kuroda Y. (2022). Hypothermia for Patients Requiring Evacuation of Subdural Hematoma: A Multicenter Randomized Clinical Trial. Neurocrit. Care.

[10] Clifton G.L., Coffey C.S., Fourwinds S., Zygun D., Valadka A., Smith K.R., Frisby M.L., Bucholz R.D., Wilde E.A., Levin H.S. (2012). Early Induction of Hypothermia for Evacuated Intracranial Hematomas: A Post Hoc Analysis of Two Clinical Trials. J. Neurosurg..

[11] Chen H., Wu F., Yang P., Shao J., Chen Q., Zheng R. (2019). A Meta-Analysis of the Effects of Therapeutic Hypothermia in Adult Patients with Traumatic Brain Injury. Crit. Care.

[12] Watson H.I., Shepherd A.A., Rhodes J.K.J., Andrews P.J.D. (2018). Revisited: A Systematic Review of Therapeutic Hypothermia for Adult Patients Following Traumatic Brain Injury. Crit. Care Med..

[13] Crompton E.M., Lubomirova I., Cotlarciuc I., Han T.S., Sharma S.D., Sharma P. (2017). Meta-Analysis of Therapeutic Hypothermia for Traumatic Brain Injury in Adult and Pediatric Patients. Crit. Care Med..

[14] Carney N., Totten A.M., O’Reilly C., Ullman J.S., Hawryluk G.W.J., Bell M.J., Bratton S.L., Chesnut R., Harris O.A., Kissoon N. (2017). Guidelines for the Management of Severe Traumatic Brain Injury, Fourth Edition. Neurosurgery.

[15] Suehiro E., Koizumi H., Kunitsugu I., Fujisawa H., Suzuki M. (2014). Survey of Brain Temperature Management in Patients with Traumatic Brain Injury in the Japan Neurotrauma Data Bank. J. Neurotrauma.

[16] England T.N. (2002). Mild Therapeutic Hypothermia to Improve the Neurologic Outcome after Cardiac Arrest. N. Engl. J. Med..

[17] Bernard S.A., Gray T.W., Buist M.D., Jones B.M., Silvester W., Gutteridge G., Smith K. (2002). Treatment of Comatose Survivors of Out-of-Hospital Cardiac Arrest with Induced Hypothermia. N. Engl. J. Med..

[18] Peberdy M.A., Callaway C.W., Neumar R.W., Geocadin R.G., Zimmerman J.L., Donnino M., Gabrielli A., Silvers S.M., Zaritsky A.L., Merchant R. (2010). Part 9: Post-Cardiac Arrest Care: 2010 American Heart Association Guidelines for Cardiopulmonary Resuscitation and Emergency Cardiovascular Care. Circulation.

[19] Nielsen N., Wetterslev J., Cronberg T., Erlinge D., Gasche Y., Hassager C., Horn J., Hovdenes J., Kjaergaard J., Kuiper M. (2013). Targeted Temperature Management at 33 °C versus 36 °C after Cardiac Arrest. N. Engl. J. Med..

[20] Callaway C.W., Donnino M.W., Fink E.L., Geocadin R.G., Golan E., Kern K.B., Leary M., Meurer W.J., Peberdy M.A., Thompson T.M. (2015). Part 8: Post-Cardiac Arrest Care: 2015 American Heart Association Guidelines Update for Cardiopulmonary Resuscitation and Emergency Cardiovascular Care. Circulation.

[21] Dankiewicz J., Cronberg T., Lilja G., Jakobsen J.C., Levin H., Ullén S., Rylander C., Wise M.P., Oddo M., Cariou A. (2021). Hypothermia versus Normothermia after Out-of-Hospital Cardiac Arrest. N. Engl. J. Med..

[22] Wyckoff M.H., Greif R., Morley P.T., Ng K.C., Olasveengen T.M., Singletary E.M., Soar J., Cheng A., Drennan I.R., Liley H.G. (2022). 2022 International Consensus on Cardiopulmonary Resuscitation and Emergency Cardiovascular Care Science with Treatment Recommendations: Summary from the Basic Life Support; Advanced Life Support; Pediatric Life Support; Neonatal Life Support; Education, Implementation, and Teams; and First Aid Task Forces. Circulation.

[23] Chen C., Ma T.Z., Wang L.N., Wang J.J., Tu Y., Zhao M.L., Zhang S., Sun H.T., Li X.H. (2016). Mild Hypothermia Facilitates the Long-Term Survival of Newborn Cells in the Dentate Gyrus after Traumatic Brain Injury by Diminishing a pro-Apoptotic Microenvironment. Neuroscience.

[24] Wang C.F., Zhao C.C., He Y., Li Z.Y., Liu W.L., Huang X.J., Deng Y.F., Li W.P. (2019). Mild Hypothermia Reduces Endoplasmic Reticulum Stress-Induced Apoptosis and Improves Neuronal Functions after Severe Traumatic Brain Injury. Brain Behav..

[25] Smith S.L., Hall E.D. (1996). Mild Pre- and Posttraumatic Hypothermia Attenuates Blood-Brain Barrier Damage Following Controlled Cortical Impact Injury in the Rat. J. Neurotrauma.

[26] Polderman K.H. (2009). Mechanisms of Action, Physiological Effects, and Complications of Hypothermia. Crit. Care Med..

[27] Vassilieff N., Rosencher N., Sessler D.I., Conseiller C. (1995). Shivering Threshold during Spinal Anesthesia Is Reduced in Elderly Patients. Anesthesiology.

[28] Perman S.M., Bartos J.A., Del Rios M., Donnino M.W., Hirsch K.G., Jentzer J.C., Kudenchuk P.J., Kurz M.C., Maciel C.B., Menon V. (2023). Temperature Management for Comatose Adult Survivors of Cardiac Arrest: A Science Advisory from the American Heart Association. Circulation.

[29] Flynn L.M.C.C., Rhodes J., Andrews P.J.D.D., Al F.E.T. (2015). Therapeutic Hypothermia Reduces Intracranial Pressure and Partial Brain Oxygen Tension in Patients with Severe Traumatic Brain Injury: Preliminary Data from the Eurotherm3235 Trial. Ther. Hypothermia Temp. Manag..

[30] Sahuquillo J., Pérez-Bárcena J., Biestro A., Zavala E., Merino M.A., Vilalta A., Poca M.A., Garnacho A., Adalia R., Homar J. (2009). Intravascular Cooling for Rapid Induction of Moderate Hypothermia in Severely Head-Injured Patients: Results of a Multicenter Study (IntraCool). Intensive Care Med..

[31] Qiu W., Zhang Y., Sheng H., Zhang J., Wang W., Liu W., Chen K., Zhou J., Xu Z. (2007). Effects of Therapeutic Mild Hypothermia on Patients with Severe Traumatic Brain Injury after Craniotomy. J. Crit. Care.

[32] Zhi D., Zhang S., Lin X. (2003). Study on Therapeutic Mechanism and Clinical Effect of Mild Hypothermia in Patients with Severe Head Injury. Surg. Neurol..

[33] Milde L.N. (1992). Clinical Use of Mild Hypothermia for Brain Protection: A Dream Revisited. J. Neurosurg. Anesthesiol..

[34] Hawryluk G.W.J., Aguilera S., Buki A., Bulger E., Citerio G., Cooper D.J., Arrastia R.D., Diringer M., Figaji A., Gao G. (2019). A Management Algorithm for Patients with Intracranial Pressure Monitoring: The Seattle International Severe Traumatic Brain Injury Consensus Conference (SIBICC). Intensive Care Med..

[35] Hui J., Feng J., Tu Y., Zhang W., Zhong C., Liu M., Wang Y., Long L., Chen L., Liu J. (2021). Safety and Efficacy of Long-Term Mild Hypothermia for Severe Traumatic Brain Injury with Refractory Intracranial Hypertension (LTH-1): A Multicenter Randomized Controlled Trial. EClinicalMedicine.

[36] Léger M., Frasca D., Roquilly A., Seguin P., Cinotti R., Dahyot-Fizelier C., Asehnoune K., Le Borgne F., Gaillard T., Foucher Y. (2022). Early Use of Barbiturates Is Associated with Increased Mortality in Traumatic Brain Injury Patients from a Propensity Score-Based Analysis of a Prospective Cohort. PLoS ONE.

[37] Kajiwara S., Hasegawa Y., Negoto T., Orito K., Kawano T., Yoshitomi M., Sakata K., Takeshige N., Yamakawa Y., Jono H. (2021). Efficacy of a Novel Prophylactic Barbiturate Therapy for Severe Traumatic Brain Injuries: Step-down Infusion of a Barbiturate with Normothermia. Neurol. Med. Chir..

[38] Bao L., Chen D., Ding L., Ling W., Xu F. (2014). Fever Burden Is an Independent Predictor for Prognosis of Traumatic Brain Injury. PLoS ONE.

[39] Rosengart A.J., Schultheiss K.E., Tolentino J., Macdonald R.L. (2007). Prognostic Factors for Outcome in Patients with Aneurysmal Subarachnoid Hemorrhage. Stroke.

[40] Bray J.E., Stub D., Bloom J.E., Segan L., Mitra B., Smith K., Finn J., Bernard S. (2017). Changing Target Temperature from 33 °C to 36 °C in the ICU Management of Out-of-Hospital Cardiac Arrest: A before and after Study. Resuscitation.

[41] Greer D.M., Funk S.E., Reaven N.L., Ouzounelli M., Uman G.C. (2008). Impact of Fever on Outcome in Patients with Stroke and Neurologic Injury: A Comprehensive Meta-Analysis. Stroke.

[42] Kinoshita K., Chatzipanteli K., Alonso O.F., Howard M., Dietrich W.D. (2002). The Effect of Brain Temperature on Hemoglobin Extravasation after Traumatic Brain Injury. J. Neurosurg..

[43] Suehiro E., Fujisawa H., Ito H., Ishikawa T., Maekawa T. (2009). Brain Temperature Modifies Glutamate Neurotoxicity In Vivo. J. Neurotrauma.

[44] Ginsberg M.D., Sternau L.L., Globus M.Y., Dietrich W.D., Busto R. (1992). Therapeutic Modulation of Brain Temperature: Relevance to Ischemic Brain Injury. Cerebrovasc. Brain Metab. Rev..

[45] Jiang J.Y., Lyeth B.G., Kapasi M.Z., Jenkins L.W., Povlishock J.T. (1992). Moderate Hypothermia Reduces Blood-Brain Barrier Disruption Following Traumatic Brain Injury in the Rat. Acta Neuropathol..

[46] Young A.B., Ott L.G., Beard D., Dempsey R.J., Tibbs P.A., McClain C.J. (1988). The Acute-Phase Response of the Brain-Injured Patient. J. Neurosurg..

[47] Picetti E., Oddo M., Prisco L., Helbok R., Taccone F.S. (2019). A Survey on Fever Monitoring and Management in Patients With Acute Brain Injury: The SUMMA Study. J. Neurosurg. Anesthesiol..

[48] Andrews P.J.D., Sinclair H.L., Rodriguez A., Harris B.A., Battison C.G., Rhodes J.K.J., Murray G.D., Eurotherm3235 Trial Collaborators (2015). Hypothermia for Intracranial Hypertension after Traumatic Brain Injury. N. Engl. J. Med..

[49] Cooper D.J., Nichol A.D., Bailey M., Bernard S., Cameron P.A., Pili-Floury S., Forbes A., Gantner D., Higgins A.M., Huet O. (2018). Effect of Early Sustained Prophylactic Hypothermia on Neurologic Outcomes among Patients with Severe Traumatic Brain Injury. JAMA-J. Am. Med. Assoc..

[50] Zhao Q.J., Zhang X.G., Wang L.X. (2011). Mild Hypothermia Therapy Reduces Blood Glucose and Lactate and Improves Neurologic Outcomes in Patients with Severe Traumatic Brain Injury. J. Crit. Care.

[51] Badjatia N., Strongilis E., Gordon E., Prescutti M., Fernandez L., Fernandez A., Buitrago M., Schmidt J.M., Ostapkovich N.D., Mayer S.A. (2008). Metabolic Impact of Shivering during Therapeutic Temperature Modulation: The Bedside Shivering Assessment Scale. Stroke.

[52] Kuroda Y. (2016). Neurocritical Care Update. J. Intensive Care.

[53] Norisue Y., Fujimoto Y., Nakagawa K. (2018). Preliminary Guideline- and Pathophysiology-Based Protocols for Neurocritical Care. J. Intensive Care.

[54] Maegele M., Schöchl H., Menovsky T., Maréchal H., Marklund N., Buki A., Stanworth S. (2017). Coagulopathy and Haemorrhagic Progression in Traumatic Brain Injury: Advances in Mechanisms, Diagnosis, and Management. Lancet Neurol..

[55] Hifumi T., Kuroda Y., Kawakita K., Yamashita S., Oda Y., Dohi K., Maekawa T. (2017). Therapeutic Hypothermia in Patients with Coagulopathy Following Severe Traumatic Brain Injury. Scand. J. Trauma. Resusc. Emerg. Med..

[56] Quine E.J., Murray L., Trapani T., Cooper D.J. (2021). Thromboelastography to Assess Coagulopathy in Traumatic Brain Injury Patients Undergoing Therapeutic Hypothermia. Ther. Hypothermia Temp. Manag..

[57] Robba C., Graziano F., Rebora P., Elli F., Giussani C., Oddo M., Meyfroidt G., Helbok R., Taccone F.S., Prisco L. (2021). Intracranial Pressure Monitoring in Patients with Acute Brain Injury in the Intensive Care Unit (SYNAPSE-ICU): An International, Prospective Observational Cohort Study. Lancet Neurol..

[58] Forcione M., Yakoub K.M., Chiarelli A.M., Perpetuini D., Merla A., Sun R., Sawosz P., Belli A., Davies D.J. (2020). Dynamic Contrast-Enhanced near-Infrared Spectroscopy Using Indocyanine Green on Moderate and Severe Traumatic Brain Injury: A Prospective Observational Study. Quant. Imaging Med. Surg..

